# The Kumagai Method: Feeding Techniques Using the Pigeon Baby Cleft Palate Bottle

**DOI:** 10.3390/nursrep14040199

**Published:** 2024-09-30

**Authors:** Shingo Ueki, Yukari Kumagai, Yumi Hirai, Eri Nagatomo, Shoko Miyauchi, Takuro Inoue, Qi An, Eri Tashiro, Junko Miyata

**Affiliations:** 1 Department of Health Sciences, Faculty of Medical Sciences, Kyushu University, Fukuoka 812-8582, Japan; nagatomo.eri.018@m.kyushu-u.ac.jp (E.N.); tashiro.eri.084@m.kyushu-u.ac.jp (E.T.); miyata.junko.789@m.kyushu-u.ac.jp (J.M.); 2 Department of Nursing, Osaka University Dental Hospital, Osaka 565-0871, Japan; kumagai-y@office.osaka-u.ac.jp (Y.K.); hirai-yu@office.osaka-u.ac.jp (Y.H.); 3 Department of Advanced Information Technology, Faculty of Information Science and Electrical Engineering, Kyushu University, Fukuoka 819-0395, Japan; miyauchi@ait.kyushu-u.ac.jp; 4 Department of Informatics, Graduate School of Information Science and Electrical Engineering, Kyushu University, Fukuoka 819-0395, Japan; inoue.takuro.882@s.kyushu-u.ac.jp; 5 Department of Human and Engineered Environmental Studies, Graduate School of Frontier Sciences, The University of Tokyo, Chiba 277-8563, Japan; anqi@robot.t.u-tokyo.ac.jp; 6 Department of Pediatric Surgery, Graduate School of Medical Sciences, Kyushu University, Fukuoka 812-8582, Japan

**Keywords:** cleft lip, cleft palate, child, feeding difficulties, qualitative research

## Abstract

Background/Objectives: This study aimed to identify the P-bottle feeding techniques systematically organized by Ms. Kumagai, an expert in nursing care for children with a cleft lip and/or palate (CLP), which were developed as she gained expertise in feeding affected children. Methods: We recruited three nurses who had mastered the Kumagai method for feeding with a P-bottle. Through analysis of participants’ voices and videos during interviews, we focused on aspects such as dealing with a closed mouth, inserting the nipple in cases of unilateral and bilateral CLP, dealing with the child’s movements after insertion, and key considerations when squeezing the bottle. Results: The interview analyses revealed numerous techniques used by nurses to manage the difficulties encountered while feeding children, ensuring successful provision of nourishment. Specifically, the nurses employed techniques such as placing the nipple along the midline of the child’s tongue and varying the application of force on the nipple depending on the cleft type. The nurses reported that the objectives of these techniques were to prevent ulcer formation and encourage the use of the tongue, simulating original feeding movements. Conclusions: We explored feeding techniques and the management of associated challenges. Our results suggest that the “Kumagai Method” could be valuable in improving feeding practices.

## 1. Introduction

Cleft lip and/or palate (CLP) is one of the most prevalent congenital facial malformations. A systematic review reported that the global prevalence of CLP was 10.8 cases per 1000 live births [1]. The challenges associated with CLP are multifaceted, encompassing concerns related to feeding, chewing, articulation, and facial esthetics [2]. Among these challenges, feeding difficulties, which are present in 35–67% of children with CLP [3,4], bear considerable significance, and are attributed to factors such as shortened sucking motions, an accelerated sucking pace, an elevated suck–swallow ratio, and an inability to establish negative pressure within the oral cavity [5]. Furthermore, the lack of muscular coordination within the oral region, including inadequate swallowing function and suboptimal sucking patterns, adds to the complexity of the situation in children affected by CLP [6]. Regrettably, feeding difficulties arising from these functional irregularities often translate to inadequate weight gain and even malnutrition [7,8,9]. Notably, the timing of the first surgical intervention for cleft lip repair is based on the often-quoted “rule of tens,” which involves “weight of 10 pounds, hemoglobin of 10 g/dL, and age > 10 weeks” [10]. However, the confluence of feeding challenges faced by children with CLP can impede their ability to attain sufficient weight gain within the prescribed timeframe before surgical intervention, potentially causing delays in the surgical schedule [11].

Breastfeeding is known to have various benefits [12,13] and is also recommended for children with CLP [14]. The World Health Organization (WHO) recommends that infants should be fully breastfed for the first six months and has presented a statement entitled “Ten Steps to Successful Breastfeeding” with recommendations for healthcare facilities worldwide [15]. Some tips for breastfeeding were reported, such as “football/twin feeding style with the infant in the semi-upright position,” “occluding the cleft with a thumb or finger,” “supporting the infant’s cheeks to decrease the width of the cleft,” “positioning the breast toward the side of the palate that has the most intact bone,” “dancer hand position, which supports the infant’s chin to stabilize the jaw during sucking,” “releasing breast milk into the infant’s mouth,” “placing the nipple downward onto the tongue,” and “taking feeding pauses by removing the breast to allow the infant to breathe” [6,16]. However, most infants with a cleft palate cannot drink enough from the breast directly, necessitating the use a supplementary bottle or cup [16]. Moreover, a recent study has shown that some parents of infants with CLP prefer mixed breastfeeding and bottle-feeding [17]. Therefore, it is necessary to consider the appropriate ways of providing sufficient amounts of milk to infants with CLP other than breastfeeding.

Various special feeding devices are available for children with CLP who cannot create negative oral pressure [14]. The Pigeon Baby Cleft Palate Bottle (P-bottle), characterized by its squeezable design, is a widely adopted option. The P-bottle’s nipple features a one-way valve, a Y-cut tip, and an air vent [16]. This bottle was first developed in Japan, and has now gained global usage. A recent Japanese study revealed that approximately 60% of nurses use the P-bottle for feeding children with CLP, applying a variety of feeding techniques [18]. However, contradictory techniques such as “inserting the nipple to not touch the cleft” and “closing the cleft using the nipple to create negative pressure in the oral cavity” were identified. This study concluded that feeding techniques lacked standardization. Notably, most nurses who participated in this study cared for less than five CLP patients annually [18]. Therefore, we consider that these results mostly included inadequate feeding techniques employed by less experienced nurses. Certain studies reported that even with special feeding bottles, children with CLP fed less than healthy children [19], and that simply using special bottles did not provide substantial benefits to the child’s growth [20]. These findings indicate the need to elucidate effective strategies for handling specialized bottles, especially the P-bottle.

Although reviews have investigated the techniques used for feeding bottles [16,21,22,23], the included studies largely focused on the above-mentioned techniques for breastfeeding and were not specific to the P-bottle. Moreover, infants who have difficulty feeding often exhibit a refusal response, turning their face away and pushing the nipple out with their tongues, because they cannot accept feeding. Therefore, it is necessary to know not only how to use the bottle, but also how to respond to the child’s movement patterns. A recent scoping review also reported that existing resources, including guidelines for managing the challenges associated with CLP, were inadequate [24], and developing specific feeding techniques is an important issue. Therefore, the purpose of the present study was to identify P-bottle feeding techniques performed by nurses who were experts in feeding children with CLP and feeding difficulties.

## 2. Materials and Methods

### 2.1. Study Design

The present study employed a qualitative, descriptive research design in which participants’ voices and videos were used as data. 

### 2.2. Participants

The participants were nurses working at a university-affiliated dental hospital in Japan. The hospital, equipped with a CLP center, conducts approximately 400 CLP-related surgeries annually. Consequently, since children with severe CLP from across Japan visit the hospital, this facility frequently deals with cases of feeding difficulties. Additionally, the hospital provides educational support, including on-site feeding guidance, upon request from nearby obstetric hospitals. The head nurse, Ms. Kumagai, who has worked at the hospital for several years, has independently refined feeding techniques, resulting in the name “the Kumagai Method”. This method involves two approaches, viz., using a narrow-long nipple and a P-bottle. We have already published the techniques using the narrow-long nipple [25]. The present study was designed to elucidate the P-bottle based techniques. 

To acquire proficiency in the Kumagai Method, the hospital conducts specialized training sessions internally. However, only a few participants pass these training sessions. We invited Ms. Kumagai to participate in this study and asked her to provide us with recommendations for nurses who had obtained certification in mastering the Kumagai method with the P-bottle. The researcher (S.U.) provided research explanations to these nurses, and upon obtaining consent, enrolled them as research participants.

### 2.3. Data Collection

In September 2022, participants were equipped with recording microphones and asked to stand in front of a video camera. A semi-structured, in-depth interview was conducted in Japanese to gather information on the following: dealing with a closed mouth, inserting the nipple in cases of unilateral CLP, inserting the nipple in cases of bilateral CLP, dealing with the child’s movements after insertion, and points to note when squeezing the bottle. The “child’s movements that should be dealt with” were determined based on the following factors: intense crying, swinging the body widely, pushing out the nipple with the tongue, licking the nipple with the tongue without sucking, pushing up the nipple strongly with the tongue, weak sucking, and spitting out milk (a lot or a little). These movements were confirmed with the participants in advance as undesirable movements that commonly occur in children with feeding difficulties, and were defined before commencing the study.

Participants were requested to demonstrate feeding movements while holding a child doll with an incision in the oral cavity, to simulate CLP, and simultaneously provide verbal explanations. Recordings were made through the microphone and video camera. The collected audio and video files constituted the data for this study.

The interviewer was a researcher (S.U.) with experience as a pediatric nurse in feeding children with CLP, but who was ignorant of the Kumagai method. Therefore, he proceeded with the interview while checking the meaning of each word said by the participant.

### 2.4. Analysis

We used thematic analysis based on interpretivism [26]. This method is used to identify and report patterns (themes) obtained from interviews. First, the audio data were translated into written text, and the information provided by movements in the video data were added. The text was then mailed to each participant and checked for corrections or additions. After reading to understand the meaning, each technique was intercepted and coded. The codes were grouped by semantic content. The techniques were reviewed among the authors to ensure that the content of the techniques was understandable and that the different techniques were clearly distinguishable. The analysis was conducted by a researcher who specializes in pediatric nursing and has experience in feeding with a P-bottle. After analysis, the results were returned to the participants again for perusal to ensure that there were no discrepancies in each participant’s intended meaning, which achieved investigator triangulation and enhanced the validity of the findings. The results were confirmed again by researchers and participants six months after the analysis was completed to ensure reliability.

### 2.5. Ethical Consideration

The present study was conducted in accordance with the International Committee of Medical Journal Editors guidelines, after obtaining ethical review approval and clinical research registration at Kyushu University. We explained the purpose and methods of the study to the participants and obtained their signatures on a consent form. The participants were informed that participation was voluntary and that they could withdraw at any time. Data collection was conducted in a private room in the hospitals to ensure privacy. In addition, data were managed by serial numbers to ensure that individuals could not be identified.

## 3. Results

The three nurses who had mastered the Kumagai Method had 19, 28, and 36 years nursing experience, respectively. The interview durations were 17.13, 18.78, and 24.62 min each.

### 3.1. Dealing with a Closed Mouth

Sometimes children with CLP will not open their mouth even when the nipple is close to their mouth. Three techniques were mentioned for dealing with such a situation: waking the children up with daily care, stimulating the mouth area with fingers or nipples, and lowering the lower jaw with using fingers.

I encourage the child’s sucking reflex and opening the mouth by stimulating their lips with a nipple. It is not a good idea to wait all the time without doing anything or forcing the nipple into the mouth (No.2).

### 3.2. Inserting the Nipple

The correct and incorrect methods of nipple insertion are listed in Table 1. 

It is not enough to simply put the nipple in the mouth. There is a technique to enable children with feeding difficulties to effectively use their tongues to be fed milk. By inserting the nipple straight down the middle against the tongue, the child can wrap the nipple with the tongue. The participant nurses intended to promote learning original feeding movements. The nipple of the P-bottle is shaped like a cylinder, and the depth of insertion is also important.

Since the nipple is longer than a normal nipple, it is not inserted very deeply. To avoid inducing the gag reflex, do not insert the nipple all the way to the base of the nipple. The nipple is placed in the middle of the tongue so that the child wraps their tongue around the nipple, allowing the tongue to be used as the child could learn to move the tongue for original suckling (No. 2).

**Figure 1:**
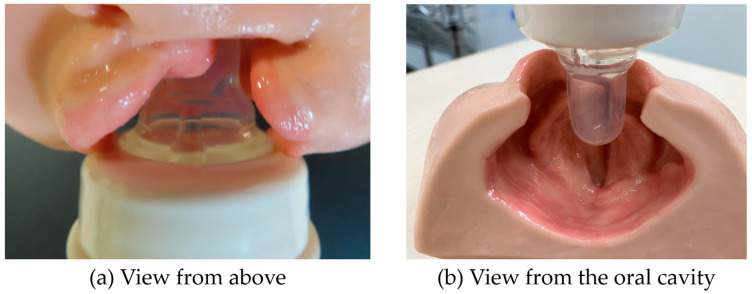
Correct position of the nipple for children with unilateral cleft lip and palate. (**a**) When viewed from above, only half the nipple is visible through the cleft. (**b**) Only half the nipple is in contact with the palate.

In the case of unilateral CLP, the participants called this maneuver “Hikkake-Nomashi” (a Japanese word devised by nurses, which means feeding while hooking the nipple onto the palate), a method that allowed only half of the nipple to be in contact with the palate area, whereas the other half remained untouched, such that the nipple was overhanging the palate (Figure 1). By employing this method, the child can squeeze half of the nipple with the tongue to release milk. The other half of the nipple is not compressed; however, the nipple does not enter the cleft, thus avoiding damage to the mucous membranes and ulcer formation.

As the child tends to bring the nipple into the cleft with their tongue, I keep the nipple overhanging a little to the palatal side to keep the nipple from getting stuck in the cleft and hold the nipple in the middle of tongue (No.1).

By placing the nipple slightly against the palate, half of the pressure to the nipple escapes into the cleft. Only half of the nipple gets the pressure to drink. As the situation continues, the child will put the tongue straight up. The child will no longer try to enter the cleft and will not have to apply force in the direction opposite to the cleft. The incorrect way is to leave the nipple as it enters the cleft. Although you feel as if the child drinks well, the nipple is, in fact, not applying any pressure with their tongue at all. In this way, the milk is not released, and the child does not drink as much as they should (No.2).

**Figure 2:**
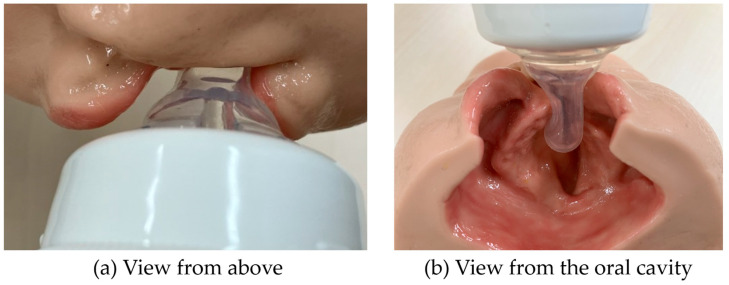
Incorrect positioning of the nipple for children with unilateral cleft lip and palate. (**a**) When viewed from above, the nipple is not visible. (**b**) The nipple is directed toward the cleft.

If the nipple goes toward the cleft (Figure 2), it will create an ulcer on the mucous membrane, so I do not think it should be done in such a way that the nipple goes in there (No.3).

In the case of bilateral CLP, there is no palate against which the nipple can be applied; hence, the nurses need to feed the child while pushing the face of the nipple in the direction of the tongue. In such cases, the pressure exerted by the child by only pushing up with the tongue is not sufficient to dispense enough milk. Therefore, the nurse has to squeeze the bottle while the child sucks to allow them to drink the milk and to prevent the child from getting tired.

In the case of bilateral CLP, lightly press the nipple toward the tongue as the nipple cannot be hooked onto the palate, so as to inhibit the child from raising the tongue up too much. Adjust the milk flow by squeezing the bottle; the bottle should be squeezed more forcefully than for children with unilateral CLP. The timing should match the child’s sucking. In the case of bilateral CLP, the key is to let the child feel that they are drinking from their own sucking (No.4).

### 3.3. Squeezing the Bottle

The P-bottles are soft and can be squeezed to allow milk to flow out. However, the participants appeared to adjust the timing and pressure and not squeeze continuously, ensuring that the child could suck carefully (Table 2).

If I squeeze the bottle too fast, the child may choke; thus, we adjust the amount of milk while looking at whether the child is swallowing properly and not choking (No.3).

Squeezing in time with the child’s breathing and sucking is a very important technique. If I squeeze the bottle at my own pace, without matching the child’s breathing and sucking, it may cause choking. Another reason is that the child may learn to drink without sucking, which leads to not sucking (No.2).

### 3.4. Dealing with Various Child Movements after Insertion

A child with feeding difficulties may exhibit a variety of movements, as shown in Table 3. The first thing that the participants would do in response to the child’s movements was to “re-hold” the child. If the child did not stop the movements even after that, the participants would take action, as shown in Table 3. 

If the child “swings the body wide,” “pushes the nipple out with the tongue,” or “licks the nipple with the tongue without sucking,” the participants assessed the situation as resistance to feeding and removed the nipple temporarily.

If the child is quiet and then suddenly arching, I think the nipple might be in a painful place. Thus, I check if I am inserting the nipple correctly and will re-insert again (No.1).

Some children exhibit a tendency to strongly force the tongue to rise and tend to move the nipple into the cleft, even if the participants attempt to perform “Hikkake-Nomashi.” In this case, the participants maintain the position of the nipple on the midline of the tongue and squeeze the bottle more to allow the child to focus on swallowing, resulting in the use of appropriate force to raise the tongue.

If the child’s tongue raising is strong, I increase the flow rate of milk. If I am defeated by the child’s force of tongue raising and the nipple enters the cleft, an ulcer is formed. Furthermore, when the nipple enters the cleft, the nipple is not compressed sufficiently and the child’s force of raising up the tongue becomes even stronger. This strong tongue raising is a response to wanting more milk, so milk flow needs to be increased. By increasing the bottle squeezing, the child will be able to drink the amount they want to drink. As a result, the force of the tongue raising will become moderate (No.2).

When the sucking is weak, it is necessary to apply a stimulus to assess if the sucking power is restored. The way to deal with this situation is to squeeze the bottle slightly and release a little milk to check if the sucking reflex would be initiated.

Even if I try to stimulate the child’s tongue or lips, the sucking reflex still does not occur, I squeeze slightly. I look to check if sucking occurs before swallowing, and if it does, I adjust the force of squeezing bottle to match the force of the child’s sucking (No.2).

## 4. Discussion

The strength of the present study is that it systematically organized the detailed technical patterns that Ms. Kumagai, an expert in nursing care for children with CLP, has achieved over time. After birth, a palatal obturator is fabricated and fitted for children with CLP, and most children are able to drink without special bottle-feeding techniques [27] and achieve breastfeeding. However, it takes about a week to fabricate a palatal obturator. Meanwhile, children with CLP, who cannot create a negative pressure in the oral cavity and cannot be breastfed directly, must become accustomed to the P-bottle. If the child is not helped to drink adequate milk, they will require tube feeding or intravenous infusion, and their natural ability to feed will degenerate [28]. The Kumagai method allows more children to drink milk orally. The other issue in this study is the effectiveness of the techniques of the Kumagai method. Ms. Kumagai recently presented a practical report in Japanese demonstrating the effectiveness of this method in actual children [29]. Implementation of the Kumagai method in children with CLP with ulceration of the nasal septum resulted in reduction of the ulcers in about 80% of children, and they could also drink the target amount of milk within 20 min [29]. Further clinical studies investigating this method with a larger sample of children are warranted to scientifically prove the extent to which the Kumagai method increases feeding volume and contributes to weight gain in children with CLP and feeding difficulties, and that it decreases the frequency of ulcer development compared to other techniques.

The novelty of this study is that we clarified the direction of nipple insertion into the oral cavity (inserting the nipple to rest on the midline of the tongue), depth of insertion (not up to the base of the nipple), and timing (squeezing the bottle at the time of the child’s sucking). Furthermore, the direction of the nipple force should be changed depending on the type of CLP (unilateral or bilateral). One of the reasons for this particular direction of pushing force for nipple placement is to prevent ulceration of the nasal septum mucosa. The mucosa of the nasal septum is soft and susceptible to ulceration due to irritation [16]. In addition, children with CLP have a tendency to try to insert the nipple into the cleft with the tongue. If the nurse allows this movement and continues to feed with the nipple inserted into the cleft, the pressure of the nipple causes ulceration. Ueki et al. [18] conducted a survey and found that many nurses use the “fit the nipple into the cleft” method, which may inadvertently cause ulcers. If the Kumagai method becomes the standard for clinical feeding, it may contribute to a reduction in the occurrence of ulcers. Other studies have suggested that the nipple be inserted in a direction without the cleft palate while tilting [30]. This method would also be less likely to result in ulcer development. However, the participants in the present study have insisted that the nipple should be directed along the midline position of tongue. The reason for this is to prevent ulceration and simultaneously promote natural movements of tongue suckling. When a healthy infant feeds, the tongue undulates in a wavy motion as it squeezes the nipple [31]. By placing the nipple on the midline of tongue, the child would be able to catch the nipple firmly with the tongue. Valentim et al. reported that poor tongue habits have a significant impact on the oral environment, with ramifications for the dentition and facial structure [32]. Adherence to the Kumagai method may have a positive impact on the dentition, by averting the characteristic facial features and speech problems that commonly occur in children with CLP.

The P-bottle can be squeezed to release milk, allowing nurses to adjust the amount of milk. However, if this adjustment is not appropriate, it can cause the child to choke. Feeding requires synchronized sucking, swallowing, and breathing [33]. If the child’s sucking ability is weak, the amount and timing of releasing the milk should be adjusted so that the three aforementioned elements are in synchronicity. However, if the force of the tongue raising is strong, the nurse should increase the milk flow rate as per the present experience with the Kumagai method. This would allow the child to concentrate on swallowing, and naturally reduce the force of the tongue raising. This flow rate adjustment was one of the techniques used to correct the child’s use of their tongue.

One limitation of this study is the small same size, limited to three participants. However, these three nurses have supported many children with feeding difficulties using the “Kumagai method,” which is the result of this study. They have demonstrated that this method has enabled children with CLP to feed using the P-bottle. It would be easy to simply increase the number of participants, but it is not easy to find practitioners with exemplary technical skills. Another shortcoming is that the present findings are based only on interviews. Besides the techniques verbally recounted in the interviews, there may be other detailed techniques that these nurses use unconsciously. Moreover, demonstrations with a doll may not have replicated reality. It is necessary to observe the nursing skills of the participants in their daily nursing practice in real children and to investigate whether or not there are other techniques that have not yet been identified.

Thus, although there is still room for further study, this study evinced a pioneering result, being the first research with the potential to expand the application of the Kumagai method as a standard feeding method in the future. Recently, many videos explaining feeding in children with CLP have been disseminated on social networking services (SNS); however, the contents have low validity [34]. To promote the global adoption of the Kumagai method, we released a brief video of the method on social media prior to this study [35,36]. We are now preparing new videos with more detailed explanations based on the results of this study. Additionally, we plan to develop a smartphone application in the future. We also believe that interventional studies utilizing these tools will be necessary to facilitate skill acquisition.

The present study does not explicitly promote bottle-feeding promotion; the WHO’s 10 Steps to successful breastfeeding, revised in 2018, have added the following: “9. Counsel mothers on the use and risks of feeding bottles, teats and pacifiers” [15]. Breastfeeding has mutual benefits for the mother and child and should be the first priority. However, when this is not possible, the existence of alternative bottle-feeding techniques, such as the one detailed in this study, would provide support to mothers.

## 5. Conclusions

The three expert nurses’ methods of feeding children with CLP and feeding difficulties using a P-bottle included detailed techniques for dealing with a closed mouth before feeding, inserting the nipple, and squeezing the bottle. In particular, the nipple position was a new perspective, wherein the nipple should be placed on the line in the middle of the tongue, and the nipple should not be inserted to all the way to the base. In addition, it was found that the direction of the nipple force was changed; this means that if the child had unilateral CLP, the nipple was placed against half of the palate, and if the child had bilateral CLP, pressure was applied in the direction of the tongue. These methods had two purposes: to prevent ulcers and to help the child learn the natural tongue movement for suckling. In the future, it is desirable to discover other unconscious practices in these expert nurses’ techniques and to scientifically verify the effectiveness of this Kumagai method.

## Figures and Tables

**Table 1:** Methods of inserting a nipple to the mouth.

Correct	Incorrect
You insert the nipple straight on the middle of the tongue and keep it in that position.	You insert the nipple in the mouth up to the base of nipple and the child’s vomiting reflex is elicited. The nipple hits the mucosa of the cleft when the child launches the nipple with the tongue.
(In the case of unilateral cleft lip and palate) You should place the nipple in the middle of mouth and only half against the palate while applying force to the opposite side of the cleft even if the child’s tongue tries to insert the nipple into the cleft, so that it is only half compressed by the child’s tongue.	You fit the nipple into the cleft; therefore, the child cannot compress the nipple with the tongue sufficiently and ulcers are formed on the mucous membranes. You poke with the nipple toward the palate.
(In the case of bilateral cleft lip and palate) You press the nipple against the raised tongue as the child sucks. You squeeze the bottle at the same time the child sucks so that the child drinks in as short a time as possible.	You leave the hard side of the nipple against the mucous membranes. You do not squeeze the bottle.

**Table 2:** Squeezing the bottle.

Correct	Incorrect
You squeeze the bottle at the same time the child sucks. You gradually increase the pressure to squeeze the bottle while making sure the child does not choke.	You do not squeeze the bottle and let the child just suck. You squeeze the bottle regardless of the child’s breathing. You continuously squeeze the bottle.

**Table 3:** Techniques other than “re-holding” to deal with the child’s movement.

Cases	Remedial Steps
Swinging the body wide Pushing out the nipple with the tongue Licking the nipple with the tongue without sucking	Removing and re-inserting the nipple gently
Pushing up the nipple strongly with the tongue	Increasing the flow rate by increasing bottle squeezing
Intense crying	Squeezing the bottle slightly
Weak sucking	1st: Stimulating the tongue or lip 2nd: Squeezing the bottle slightly 3rd: Squeezing the bottle depending on how much as the child can swallow, if sucking occurs
Spitting out milk (a little)	Continuing the feeding
Spitting out milk (a lot)	Removing the nipple and allowing the child’s breathing to recover, and then resuming

## Data Availability

The data and resources utilized in the research can be obtained from the corresponding author upon reasonable request.

## Public Involvement Statement

No public involvement in any aspect of this research.

## Guidelines and Standards Statement

We report our findings based on the standards for reporting qualitative research (SRQR) guidelines [37].
